# Framing access to medicines in developing countries: an analysis of media coverage of Canada's Access to Medicines Regime

**DOI:** 10.1186/1472-698X-10-1

**Published:** 2010-01-04

**Authors:** Laura C Esmail, Kaye Phillips, Victoria Kuek, Andrea Perez Cosio, Jillian Clare Kohler

**Affiliations:** 1 Leslie Dan Faculty of Pharmacy, University of Toronto, 144 College Street, Toronto, Ontario, Canada

## Abstract

**Background:**

In September 2003, the Canadian government committed to developing legislation that would facilitate greater access to affordable medicines for developing countries. Over the course of eight months, the legislation, now known as Canada's Access to Medicines Regime (CAMR), went through a controversial policy development process and the newspaper media was one of the major venues in which the policy debates took place. The purpose of this study was to examine how the media framed CAMR to determine how policy goals were conceptualized, which stakeholder interests controlled the public debate and how these variables related to the public policy process.

**Methods:**

We conducted a qualitative content analysis of newspaper coverage of the CAMR policy and implementation process from 2003-2008. The primary theoretical framework for this study was framing theory. A total of 90 articles from 11 Canadian newspapers were selected for inclusion in our analysis. A team of four researchers coded the articles for themes relating to access to medicines and which stakeholders' voice figured more prominently on each issue. Stakeholders examined included: the research-based industry, the generic industry, civil society, the Canadian government, and developing country representatives.

**Results:**

The most frequently mentioned themes across all documents were the issues of drug affordability, intellectual property, trade agreements and obligations, and development. Issues such as human rights, pharmaceutical innovation, and economic competitiveness got little media representation. Civil society dominated the media contents, followed far behind by the Canadian government, the research-based and generic pharmaceutical industries. Developing country representatives were hardly represented in the media.

**Conclusions:**

Media framing obscured the discussion of some of the underlying policy goals in this case and failed to highlight issues which are now significant barriers to the use of the legislation. Using the media to engage the public in more in-depth exploration of the policy issues at stake may contribute to a more informed policy development process. The media can be an effective channel for those stakeholders with a weaker voice in policy deliberations to raise public attention to particular issues; however, the political and institutional context must be taken into account as it may outweigh media framing effects.

## Background

In September 2003, the Canadian government committed to developing legislation that would facilitate greater access to affordable medicines for developing countries. Over the course of eight months, the legislation, now known as Canada's Access to Medicines Regime (CAMR), went through a controversial policy development process that involved a variety of stakeholders with different interests and policy objectives.

CAMR policy debate triggered a wave of scholarship from policy, legal, and public health perspectives; however, little attention has been given to the role of the media throughout its policy development and implementation even though it was one of the major venues through which this policy debate unfolded. The purpose of this study was to examine how the media framed CAMR to determine: 1) how policy goals were conceptualized; 2) which goals were predominantly voiced; 3) which goals were silenced, and 4) which stakeholder group controlled the public debate. Results will be discussed in relation to CAMR's policy development process to infer the potential effect of framing in the media on policy formation and the final policy product. This study is a precursor to future work that will examine how the framing of policy goals relates to the agenda-setting processes within the development and implementation of CAMR.

### Global Drug Access and Policy Trade-Offs

The global inequity in access to medicines has received significant international attention over the last decade. Approximately one third of the world's population still lacks regular access to life-saving medicines[1]. Like most health system problems, the issue of drug access is complex and multi-faceted. The factors that prevent populations from accessing life-saving drugs often reflect the state of their health care systems: lack of infrastructure, human resources, financing and corruption are only some of the causes[1]. However, one issue that has received considerable attention has been the impact of patent protection on drug access.

The World Trade Organization's (WTO) Trade-related Aspects of Intellectual Property Rights Agreement (TRIPS) requires all WTO Member countries to implement minimum standards for the protection of intellectual property. The exclusive marketing rights obtained through intellectual property protection can restrict market competition and result in high drug prices[1]. The Doha Declaration, signed by the WTO Ministerial Council in November 2001, reaffirmed countries' rights to use the TRIPS safeguards to protect public health and in 2003, the World Trade Organization waived Article 31(f) of the TRIPS Agreement[2]. Consequently, countries with pharmaceutical manufacturing capacity were permitted to enact domestic legislation allowing the production and export of generic versions of patented drugs under compulsory licence to countries with insufficient or no manufacturing capacity (the "Paragraph 6 Decision").

Canada immediately responded to this decision in September 2003 and was the first country to announce that it would implement the agreement to facilitate greater access to affordable medicines for developing countries. After a lengthy legislative process, Bill C-9, now known as Canada's Access to Medicines Regime (CAMR), was passed in May 2004. Since then, CAMR has been heavily criticized by many as being too complex and burdensome to use for both generic companies and importing developing countries[3]. Indeed, results to date suggest show minimal impact: only one shipment of drugs has been produced and exported since its inception, with no sign of future shipments[3].

The newspaper media was one of the major mediums through which the policy debate unfolded. Civil society's call to the Canadian government to implement the WTO Paragraph 6 Decision [4], the Government's announcement of its intention to pass legislation [5] and the research-based industry's critical response [6] were among the many news reports that captured front page headlines in the major national print media. The legislation had a high priority with the outgoing and incoming Liberal Prime Ministers, the latter whom referred to it in his Speech to the Throne [7] and his address to the World Economic Forum [8]. Furthermore, civil society acknowledged their strategic use of the media to push their policy points[9]. Given the potential influence of the media on public opinion and on political elites' policy considerations [10], what is said in media and by who has implications on public policy.

### Media as a Framer of Policy

How problems are understood is dependent upon the language used to describe it. The concept of framing describes that process of taking a complex situation and emphasizing certain aspects over others[11]. By setting the terms of the debate, framing determines what arguments and facts are relevant and compelling. Furthermore, the development of strategic messages through framing can make "what is wrong and what needs fixing" [11] seem incredibly obvious.

The media's issue-framing holds the potential of influencing public perceptions of policy issues at large[12]. Used as a political tool the media may be driven by elite stakeholders to construct and control the dissemination of messages that serve a particular interest. Conversely, the media may also be understood as a vehicle for less powerful stakeholders to expand a scope of conflict and voice positions of contestation[13]. Ultimately, the framing and extent of coverage of a stakeholder's interests may be deemed a success if their frame becomes the guiding assumption amongst policy makers and influences policy making decisions.

This research questions how CAMR was framed during its five year inception, how policy goals were conceptualized, which goals were silenced and as a result, which stakeholder interests predominated the debate. Our research questions can only address the representation of issues through the analysis of media messages; therefore, we can only infer the potential or intended effects of newspaper coverage on the public, and therefore its potential role in the policy process.

This study involved a directed qualitative content analysis of newspaper coverage of the CAMR policy process during its first five years from 2003-2008. Framing theory guides our study with the goal of informing future work that determines how the framing of identified policy trade-offs link to the policy and decision making processes throughout the development and implementation of CAMR.

## Methods

### Data Collection

The exclusive focus of this study was print newspaper media as print newspapers remain a traditional source of news information in Canada. In 2007 three quarters of Canadians over 18+ read a printed edition of a daily newspaper on a weekly basis. Despite an emerging readership of online readers in the past several years, only 3% of newspaper readers read only online editions[14]. The average daily circulation of print newspapers in 2007 stood at 4,674,900[14].

A purposeful criterion sampling technique was used to identify and select regional newspapers and the two national Canadian newspapers, with the largest circulation rate[15]. Purposeful criterion sampling is the strategic selection of information rich cases based on the purpose and resources of a study. Given the national scope of the topic the study team believed sampling a mix of the most popular regional and national newspapers would conveniently fulfil the study intent. The ProQuest Newspaper database was used to identify newspapers which met the above criteria and a search of the newspapers websites was conducted to verify the circulation reporting. A total of eleven newspapers fit the criteria and were selected. These are listed in Table 1. The selection period for article sampling was August 2003-June 2008. This time period represented the inception of the of the proposed Bill C-9 legislation to its present use.

**Table 1:** Canadian Newspapers Searched

Rank	Newspaper	Weekly Circulation
1	*Toronto Star*	3,260,621
2	*The Globe and Mail*	2,024,320
4	*La Presse *(Montreal)	1,524,582
6	*National Post*	1,236,020
7	*Vancouver Sun*	1,030,691
8	*The Gazette *(Montreal)	974,021
9	*Ottawa Citizen*	919,931
10	*Winnipeg Free Press*	885,986
12	*Edmonton Journal*	873,754
15	*The Chronicle-Herald *(Halifax)	736,371
19	*The Times-Colonist *(Victoria)	502,675

Articles were selected according to their inclusion of one or more of the following search terms. These terms capture and represent core elements of the global medicine access debate and the framing of Canada's Access to Medicines Regime: access to medicine(s); medicine(s); access to essential medicine(s); access to drug(s); drug access, access to pharmaceuticals; pharmaceutical access; access to essential pharmaceuticals; Bill C-9, Canadian Access to Medicines Regime, CAMR and Jean Chrétien Pledge to Africa. This sample was augmented with articles that were already acquired by the research group as part of a larger study but were not captured within our search. Given the potential for duplication, the articles were screen to avoid double reporting. A total of 90 unique articles were identified and selected for inclusion in our analysis.

### Data Analysis

A directed qualitative content analysis technique was used to identify and analyze themes in the data[16,17]. The technique uses existing theory and literature to inform the coding scheme while permitting new concepts that appear in the data to be incorporated within the existing framework. To permit a more explicit view of the patterns that led to our associations and conclusions, we counted the number of passages coded in the categories[18]. The content and meaning of these categories were then examined and analyzed.

The concepts that informed the analytical coding scheme were informed by the doctoral thesis research of one of the authors (LE), which investigates a similar topic. A team of four researchers with combined expertise in drug policy and law further developed the coding scheme and analyzed the data. To ensure the validity and reliability of the coding scheme and procedure, [17] eight articles were pulled at three different time points and distributed across the four researchers. All articles were coded separately and then the researchers met to compare results. Each article was reviewed against the codebook, discrepancies were discussed and consensus was gathered to revise, add or remove themes, and clarify existing codebook themes. This procedure was repeated three times at which point there was full agreement on the codebook and its definitions.

The codebook captured the themes and concepts including but not exhaustive: intellectual property; trade agreements and obligations; drug affordability; quality, safety and regulatory approval; innovation; market competition; industry profits; aid; development; domestic economy; corruption and transparency; and CAMR-specific themes. The articles were divided amongst the four researchers who conducted all the coding. Codes were entered into NVivo 8 to facilitate analysis and summarization of the contents of the themes. NVivo is a qualitative data analysis software package that facilitates the organization and analysis of non-numerical or text-based data[19].

Individual themes were re-analyzed for which stakeholders' 'voice' figured more prominently on the issue. We defined the actor's voice as whenever an actor is quoted or paraphrased. Stakeholders examined included: the research-based industry, the generic industry, civil society organizations and prominent individuals, the Canadian government, other governments and international governmental organizations.

## Results

### The Story

Media coverage on Canada's implementation of the Paragraph 6 Decision started on September 23, 2003 with reporting of Stephen Lewis' call to G7 nations to step up and amend their patent laws to facilitate the production and export of drugs to developing countries. He urged Canada in particular given its generic industry and the Liberal government's foreign policy commitment to fighting HIV/AIDS.

"Canadian Stephen Lewis...said in an interview that a G7 nation must step forward immediately with a patent initiative to break the logjam preventing the mass production and export of up to $1-billion (U.S.) a year of life-saving drugs, mostly for Africa" [4].

Shortly after, the Liberal government announced that it would soon introduce legislation to facilitate the WTO Decision. Cabinet Ministers and government officials were quoted with much self-congratulatory praise, speaking of setting a precedent, blazing the trail and encouraging other countries to follow suit.

"Prime Minister Jean Chrétien said Canada is making history by following through on a World Trade Organization initiative to allow patent laws to be changed so that low-cost generic versions of brand-name drugs could be shipped to developing countries"[20].

The legislation generated a flood of praise for the Canadian government from all over the world, hailing Canada as an international leader. The legislation was greeted with high hopes, framed "a major breakthrough in the international community's capacity to provide treatment and access to medicines" [21].

"Bono singing Chrétien's praises; 'Your leadership on Africa will be a legacy', Liberals' efforts to pave the way for the sale of cheap, generic-produced drugs to Africa. 'Canada way out ahead in pioneering solutions so desperately needed by the poorest and most vulnerable,' Bono wrote"[22].

Initially, the research-based industry did not join the chorus of praise, stating that the legislation would not solve a thing, hurt domestic investment and would erode patent protection. This discourse quickly subsided, however, with the industry quickly issuing its support for the legislation.

"Canada's Research-Based Pharmaceutical Companies, a lobby group for the brand-name firms, said in a press release that it recognizes Canada 'has an opportunity to show international leadership' by changing patent laws to improve access to drugs"[23].

Soon after the flood of enthusiasm, the tone of the media quickly changed having received word that the draft legislation might be problematic. Activists and the civil society community were quoted in the media as describing the legislation as "fatally flawed,"[24] potentially undermining the law and threatening Canada's chance at showing global leadership. Suggestions that the government might embarrass itself if it passes a flawed bill and would "betray those people in poor countries it purports to assist"[25].

"The new Prime Minister, Paul Martin, faces a choice: Will Canada take a strong global leadership role for the greater good of humanity or will Canada fold under the pressure of corporate pharmaceutical giants and place their profits ahead of public health?"[26]

This discourse dominated the papers from late 2003, through the parliamentary committee hearings, until it was passed on May 14, 2004. At that point, the legislation received mainly negative coverage. It was tagged as being "needlessly complicated" [27], discourages generic company participation, and was more burdensome than the WTO Paragraph 6 Decision.

"Canadian triumph turns sour: We thought we were setting an example for the world. Now we can only hope the world doesn't look too closely"[28].

The Liberal government, however, had more positive comments regarding the legislation with Liberal MP Brent St. Denis, who headed the Parliamentary committee, calling it a good model for the world.

The 2006 AIDS Conference held in Toronto marked another spike in media coverage. Much of the coverage was negative in tone, highlighting the fact that no exports had yet to be achieved by the legislation, calling for the government to review it, amend it, and also take the issue back to the WTO level.

"'We have failed lamentably,' said Lewis. 'It's almost unbelievable that two governments - one Liberal and one Conservative - can't get a single pill to Africa.'"[29].

Shortly after, the Conservative Government announced its intention to review the legislative initiative and initiated the consultation process in November 2006.

### The Issues: How did the media frame drug access in the case of CAMR?

**Figure 1:**
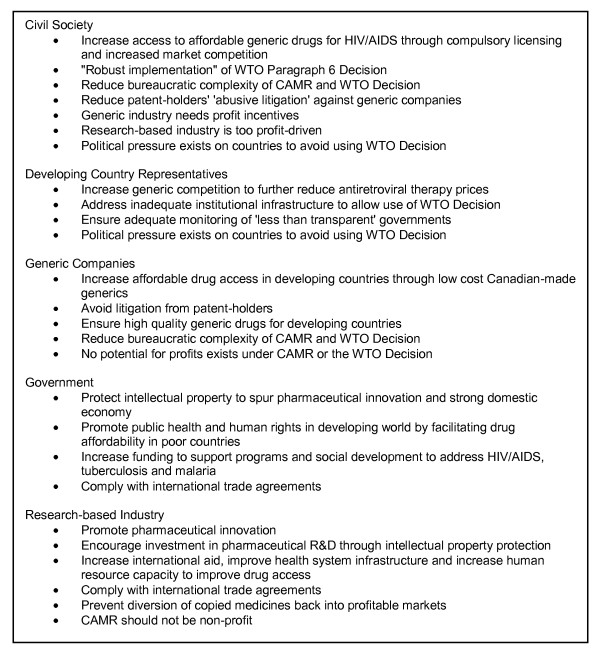
**Main Stakeholder Policy Goals and Positions as Portrayed by the Media**.

Table 2 presents the frequency with which the media discussed the major policy goals. The most frequently mentioned themes across all documents were the issues of affordability (212 paragraphs), intellectual property (198 paragraphs), trade agreements and obligations (111 paragraphs), and development (96 paragraphs). The contents of the major themes are summarized below with an explanation of which actors' voices were predominant (Additional File 1). Figure 1 provides an overview of the major policy goals and positions for each stakeholder group as portrayed by the media.

**Table 2:** Frequency of Discussion of Major Policy Goals^1^

**Aid**	43
**Corruption and Transparency**	21
**Development**	96
**Affordability**	212
**Profits**	37
**Market Competition**	32
**Domestic economy**	13
**Human Rights**	3
**Innovation**	14
**Production Costs**	7
**Procurement Practices**	8
**Quality, safety, regulatory approval**	50
**Litigation**	27
**Developing Country Pressure**	9
**Trade Agreements and Obligations**	111
**Intellectual Property**	198

^1^Frequency counts indicate number of paragraphs where the policy goal was discussed.

### Affordability

Government and civil society voices were most frequently observed on this topic. Government declared its commitment to making affordable drugs available to poor countries, with reports initially suggesting that the impact of Canada's legislative initiative could save millions of lives. The Foreign Affairs Minister wrote an opinion piece immediately prior to the legislation's announcement, linking the issue of affordability with government intervention.

"With these lives at stake, governments should responsibly intervene to ensure that medicines are available to those unable to pay market price"[21].

After the legislation was passed, voices representing government were heard less in regard to Canada's potential to contribute, saying that if the legislation simply encourages other countries to make more cheap drugs then it was fulfilling its goal.

Civil society voices were reported as emphasizing the "desperate need" for affordable medicines in the developing world, arguing that the AIDS pandemic requires a "massive inflow of affordable anti-retroviral drugs"[30]. They viewed CAMR as part of the solution.

"Canada could do much, much more to respond to the desperate need for affordable medicines in many developing countries. Most of these people died prematurely because they could not afford to buy their lives -- because medicines accepted as standard in wealthy countries are simply too expensive. Had the drugs been more affordable, many of these deaths could have been prevented"[31].

Viewed as one more source of generic AIDS drugs, civil society also viewed CAMR as key to generating market competition thereby decreasing global prices. Stephen Lewis' voice figured prominently on this topic, as with most themes emphasized by civil society. The media reported Lewis' call to the government to amend its patent law and implement the August 30, 2003 WTO Decision to allow a sustainable supply of affordable medicines, given the prospect of existing generic drugs sources in India drying up.

"India is now supplying low-cost drugs, off patent. But this is for a limited period of time, then they will have to comply with international trade laws. India also has a growing problem with AIDS, and its drugs will be needed increasingly within the country. What Canada is doing gives hope that the supply will be sustained"[32].

The research-based industry was quoted infrequently on affordability, linking the legislation to the threat of diversion of these medicines back into profitable, developed country markets. They also suggested that the issue had already been addressed, with India already supplying the developing world with AIDS medicines along with their own voluntary price reductions. The Gates Foundation echoed the industry's view that drug affordability is no longer an issue.

"Melinda Gates...said yesterday getting drug companies to lower their prices so more drugs can make it out to impoverished African nations isn't really an issue any more. 'The issue now is how we retain enough personnel in these countries to help administer and deliver the drugs on an ongoing basis,' she told reporters. 'And that cost is still very high.'"[29].

The generic industry was the least vocal on this topic, referring mainly to the low cost of their drugs and their ability to compete globally. After the legislation was in effect, they mentioned the disinterest of Canadian generic companies in producing low cost drugs under the legislation, arguing as the brand industry did that the need was already being filled by suppliers from India.

### Intellectual Property

Civil society's voice was most dominant on the issue of intellectual property (IP) and patents. They predominantly framed IP in terms of trade and as a threat to public health. Emphasis was placed on the restrictions patent laws create to making drugs accessible to developing countries, arguing that the "doors to generic drug production and export are closing fast" [32].

Lewis appeared to echo Médecins Sans Frontières' (MSF) rhetoric that patents were a "matter of life and death" [33] and framed the Canadian government as holding Africa to a lower standard, given its move in 2001 to waive patent law to produce low-cost drugs for Canadian during the Anthrax scare. He framed CAMR as a quotient of hope for eliminating IP-related barriers to drug access.

"Canada's Stephen Lewis, the United Nations envoy for HIV/AIDS in Africa, calls this 'the largest quotient of hope we've had in a long, long time.' Certainly, something had to be done. Of the 6 million who need AIDS drugs in the poor countries, barely 300,000 get them. The WTO decision came none too soon"[34].

The Liberal Government was also frequently observed on the issue of IP, framing it as innovation and knowledge, as providing incentives and funding for future R&D and for ensuring the development of new medicines for the future. One op-ed article prior to the government's announcement by Foreign Affairs Minister, Bill Graham, implied that in this particular case, drug affordability took precedence over patents.

"There are legitimate concerns that our access-to-medicines initiative might interfere with the capacity of pharmaceutical companies to fund the research and development that will produce new medicines of the future. We all benefit when intellectual property rights provide incentives for innovation. But the problem is that sick people in poor countries cannot wait until new medicines are affordable"[21].

However, government clearly said that they still valued intellectual property protection, linking it to Canada's domestic economy.

"We will continue to protect intellectual property because we believe, and [Liberal leadership front-runner] Paul Martin said last week, that innovation and knowledge is the future of Canada's economic success"[5].

There was a clear challenge for the Canadian government to enact legislation that catered to the health emergencies of poor countries while protecting intellectual property for the success of Canada's domestic economy. Ideas of crafting a precise "surgical strike" to patent laws were iterated[6]. The government's balance was framed as one between affordability and intellectual property rights, as well as international development and intellectual property rights.

"Senior government officials say they're determined to craft a precise, 'surgical strike' change to the act that would allow generic drug-makers to manufacture for export certain medicines still under patent, but which has no other impact on patent rules"[6].

But another senior official in another department said Ottawa is still not prepared to deny brand-name drug companies the right to supply medicines ahead of generic companies that would simply be copying their intellectual property[24].

The research-based industry limited their remarks on IP but was clear on their position. Initially, as seen earlier, the brand industry reacted with scathing remarks with the Director-General of the IFPMA, Harvey Bale, linking the Canadian initiative to the erosion of intellectual property rights, which would negatively impact domestic R&D investment. Bale also accused the Canadian generic industry of ulterior motives to erode patent protection.

"He cautioned that the changes contemplated in Canada will erode patent protection and scare off future research spending by biotech investors leery of changes to intellectual property right guarantees"[6].

After these initial statements, the tone of the brand industry's comments changed immediately, citing support for the legislation and saying that Canada "has an opportunity to show international leadership" [23] by changing patent laws to improve access to the drugs. The industry's position on IP was further evidenced through a letter to the editor suggesting that patents are not the barrier to drug access, citing that 95% of drugs listed on the WHO's Essential Medicines List are not protected by patents.

"Intellectual property protection is not a barrier but a facilitator to access. If intellectual property rights are not respected, new medicines may not be developed to provide better treatments and cures for diseases that impact the most vulnerable"[35].

The generic industry had virtually no comments aside from their reference to the industry's litigious nature in relation to patent infringement.

### Trade Agreements and Obligations

Civil society was, again, the most noticeable voice in this category. The key messages voiced by NGOs transformed over the life of the legislation; however, the framing remained consistent. In the early stages, most of civil society discourse reiterated calls to government to implement the WTO Paragraph 6 Decision saying that the agreement "...removes any shred of an excuse that Canada's hands are tied by our international obligations"[31]. Acknowledging that the WTO Decision was complicated on its own, they argued that if 'robustly implemented with a minimum of interference', the legislation would be worthwhile.

"Rather than creating unnecessary, additional privileges for brand-name companies, why doesn't the government just proceed with a straightforward and faithful implementation of the WTO decision from last fall?"[25].

During the time when amendments to the legislation were being proposed, civil society framed the rules embedded within the legislation as counter-productive, restrictive and an invitation to abusive litigation by patent-holders. Above all, they framed the Canadian legislation as going above and beyond what was required by the WTO Decision.

After the legislation was passed, NGOs continued to emphasize the threat of trade and intellectual property laws and the failures of the legislation. Particularly, the flaws in the Canadian legislations process, including its complexity, inefficiency and extra requirements, were at the forefront of reporting. The flaws in the system were commonly referenced back to the original WTO framework.

"At the bare minimum, said Elliott, Canada needs to get rid of the extra requirements it added. But that, he warns, would be 'sort of like tinkering around the edges' and not addressing the real problem which is the original Aug. 30, 2003 decision by the WTO to allow copies of patented drugs"[29].

Government's voice was less pronounced but still present on this issue. Canadian government officials were vocal in expressing a sense of urgency for leading a change in implementing the WTO agreement to combat pandemics such as AIDS, malaria and TB experienced in developing countries. They were hopeful that they would be a leading example and noted the possibility that their legislation could become the model.

"Canada will be the first country to introduce legislation to implement the WTO agreement. We hope that our quick response will encourage other countries to follow our example,"[20].

The remainder of the discourse in this section framed the design of their legislation in relation to the obligations and requirements of the WTO agreement. The complexity and ambiguities of the WTO agreement was also expressed, showing the Canadian government's trepidation.

"'What we're doing here, this is half symbolic and half first step,' one official said on condition of anonymity. 'We're not ready with all of the details. It's a complex scheme of royalties and applying for diseases. We're going into unknown territories, so to speak.'"[20].

The research based pharmaceutical industry's few comments framed the WTO Decision's implementation in relation to keeping future innovation and the development needs of developing countries in mind.

"The brand-name industry 'is working with the federal government to determine how the WTO decision can best be implemented, taking into account the current and future needs of developing countries, of Canada and of patients worldwide,' Mr. Elston said in the statement"[23].

The generic industry's comments were most apparent well after the legislation had passed, without effect. It blamed the complexity of the WTO agreement and also of CAMR for taking on requirements beyond what the WTO required.

"He blamed the [WTO] agreement's bureaucratic complexity. 'I remember the Indian generic association at the time saying, 'we don't think this is ever going to be used.' So far, they've been right.'"[36].

### Development

Upon announcement of the legislation, Canada was hailed as a leader in international development, receiving wide praise from around the world by figures including UN Secretary General Kofi Annan, the World Health Organization, UNICEF, Stephen Lewis, the UN's special envoy on HIV/AIDS in Africa, and rock star and international development advocate, Bono.

"I do believe we're at a moment when Canada's proven commitment and reputation can profoundly affect how we think about these issues and what is possible to achieve"[37].

Reports emphasized both the impact of HIV/AIDS on development as well as the need to address development issues to combat the pandemic, including a need for increased resources to develop the health care infrastructure and address treatment, care and prevention. Overall, a clear association was being made between development and health improvement, although its directionality shifted depending upon the messenger.

The dominant voice in this category was civil society and, in particular Stephen Lewis and MSF. Their focus was on the terrible toll of HIV/AIDS on individuals and communities, which was framed in terms of emergency and human tragedy, as well as the fact that development in Africa is paralysed by the epidemic. They also emphasized the existing global drug inequity and the need for developed countries to support Africa by making drugs available.

"In turn, economies are reeling; having already lost about 20 years off average life expectancies, countries severely affected by HIV/AIDS will lose at least 20 per cent of their GDP by 2020. As sick workers become unable to work land, and the uninfected focus on caring for sick and orphaned children, traditional agriculture is collapsing in many parts of southern Africa. Commerce is suffering as companies incur losses in the form of higher disability pensions, funeral benefits, and turnover and training costs"[21].

The government, which was less vocal, framed CAMR largely as a tool to help address the HIV/AIDS pandemic and therefore address development, with the Prime Minister and Minister of International Trade particularly supportive of the initiative. The Liberal government's initiatives and financial commitments to HIV/AIDS programming and social development initiatives were mentioned. One MP from the Conservative opposition party joined the voices in condemning the human tragedy of HIV/AIDS, citing his party's support for the legislation.

"'It is a priority for us to implement the WTO agreement that will ensure that poor countries have access to medicine to combat pandemics such as AIDS, malaria and tuberculosis,' Mr. Pettigrew said in an interview in Toronto"[33].

In contrast, the research-based industry framed development as part of the problem of drug access and in that sense development should be first addressed through improved health care infrastructure, training and delivery. They stated their position with scathing remarks upon announcement of the legislation, arguing that access to cheaper generic drugs should be at the "bottom of the totem pole" [6] of solutions and that cash should be funnelled into the infrastructure of developing countries and to the Global Fund.

"Mr. Bale questioned whether the Liberal government, even if it did introduce legislative changes, would be doing much to really help Africa. '[Stephen] Lewis is leading us all down the primrose path to a dead end,' Mr. Bale contended, saying more money for medical infrastructure in Africa, rather than yet another international supplier of cheap drugs, is what's needed to fight AIDS and other health crises"[6].

As stated earlier, Melinda Gates supported this position.

Only two passages were identified from global institutions, the WTO Director General is reported to have supported the right of poor countries to make full use of TRIPS flexibilities to counter diseases, while a research-based industry op-ed piece quoted the WHO Director General as saying that better health infrastructure and clean water are essential to effectiveness of medicines. The generic industry was notably silent on this issue.

Hardly mentioned were calls to develop manufacturing capacity in the developing world and inadequate institutional capacity to implement the WTO provisions, the latter noted by a Tanzanian government official during the 2006 AIDS Conference.

"Tanzania is not in a position to fulfill all requirements needed before importing drugs. The country is now identifying changes to its laws necessary for it to place orders with generic companies"[27].

### Aid

Overall, reports recognize that developing countries need more international assistance to fight the AIDS pandemic. The cooperation of international organizations, NGOs and developed countries on delivering this assistance beyond the distribution of drugs was noted. The media coverage of the voices of civil society, the research-based industry and government were approximately equal.

Stephen Lewis, in his capacity as UN Special Envoy, spoke of the massive inflow of aid and antiretroviral drugs required in sub-Saharan Africa. He framed this issue in terms of the West's moral deficit, criticized Canada specifically for lagging in its aid commitments and framed CAMR as a way to restore some of Canada's moral authority.

"'And,' Lewis said, 'this will help to restore Canada's position in international aid, and its moral authority. (Among the developed countries), Canada's contribution to aid has dropped from sixth to 16th place. That means there's too big a gap between rhetoric and action.'"[32].

The generic industry is reported as supporting CAMR, framing it as an aid initiative, but having a problem with its provisions for litigation.

"It's like saying, 'please come down and donate to the food bank, but you better be careful because you might end up getting sued', said Mr. Keon"[36].

The Liberal Government is reported as being enthusiastically supportive of initiative to make cheap drugs more available. Paul Martin reported to go further than that and urged other G7 countries to donate to the Global Fund, assist developing countries to win approval from WTO to import cheap drugs and to exploit CIDA's capacity.

"He [Paul Martin] urged the federal government to go even further yesterday, suggesting that Canada lobby other Group of Seven countries to join in, increase its funding to the Global Fund to fight AIDS, help developing countries win approval from the World Trade Organization to import cheap drugs, and use the Canadian International Development Agency to distribute the drugs to poor countries"[38].

The research-based industry indicated full support of the initiative, citing the need to improve access to medicines and health care systems. In an opinion piece, the industry recognizes the human toll caused by HIV/AIDS. They establish that its commitment to this cause predates CAMR, by enumerating its monetary donations, partnerships with NGOs, and capacity building in Africa.

"First of all, we recognize the untold suffering caused by HIV/AIDS and the threat it poses in the developing world. That is why research-based pharmaceutical companies globally are currently working in partnership with more than 25 international aid programs including United Nations agencies, the Bill and Melinda Gates Foundation and many others. Since 2000 alone, we have provided billions of dollars in aid and enough health interventions for 539 million people or more than two thirds of the population of sub- Saharan Africa"[39].

### Market Competition

Civil society was by far the loudest voice on this issue (in terms of strength of language, and level of coverage), arguing generally that the role of CAMR is to increase generic competition in the market for antiretroviral drugs. There was significant criticism of the research-based industry and the use of the terms "monopoly" and "monopolistic" appeared several times. Initial reports suggest that some African activists viewed CAMR as capable of producing a significant effect on drug prices.

"'For us, this is an historic occasion,' TAC chairman Zackie Achmat said. 'It's come late. It's come at a cost of many thousands of lives, but we now want to say to the drug companies, 'Let's put this behind us, and move on.'' Mr. Achmat, who is HIV-positive, now pays $74 per month for drugs (in a country where the average monthly income is $305), but the new competition could drive the price as low as $18 per month"[40].

The Liberal government's few comments supported this role, regardless of whether it leads to shipments, on the basis that "it offers an alternative drug supplier when poor countries negotiate with patented-drug makers"[41].

Civil society also criticized CAMR as giving the research-based industry the chance to block the generic competition required to bring medicines prices down under the so-called "right of refusal" clause. As such, it was a self-defeating piece of legislation. This particular provision was referred to as competitive and efficient, by the research-based industry.

"'We believe the importing countries should have options as to who can supply at the best possible price in the most timely fashion,' Jacques Lefebvre said"[42].

The remaining comments made by other stakeholders spoke of market competition under different terms. Research-based companies argued that the market "has no room for Canadian companies, since generic drugs from India and Brazil will undercut their rock-bottom prices"[43]. Generic companies seemed to support this idea, acknowledging the possibility that they may not be able to compete globally on price but would lead on quality setting a standard for the global ARV market.

### Developing Country Pressure

Civil society was the only voice that described the pressure that developing countries face in using the flexibilities in intellectual property law in trying to improve access to medicines. They described "attempts by wealthy countries" to "weaken proposals from developing countries...acting in the interests of the multinational pharmaceutical companies" during earlier rounds of WTO negotiations[31]. They argued that the WTO Paragraph 6 Decision still offers opportunities for governments to challenge countries in their attempts to use the procedure.

"MSF has argued, as well, that countries are reluctant to declare their intent to take advantage of the compulsory licence provisions because of fear of pressure from brand-name pharmaceutical companies and the United States"[27].

The Tanzanian High Commissioner was quoted in relation to this pressure as well.

"'One wouldn't expect pharmaceutical companies to be very keen about this, and we know that some countries try to protect their own domestic industry,' says Sefue. 'One can't be oblivious to the fact that there might be political pressure for countries not to follow that route -- that is a reality of the world.'"[27].

### Quality, Safety and Regulatory Approval

The generic industry's voice was prominent on the issue of drug quality and regulatory approval both before and after the legislation was passed. Early statements suggested that the Canadian generic industry's role was really to set a standard for the quality of generics AIDS drugs. During CAMR's implementation, Apotex referred to a lengthy drug development and drug approval process.

"Manufacturers will probably need 18 to 24 months to apply for approvals from Health Canada, find raw materials and set up production lines, said Jack Kay, president of Toronto-based Apotex Inc., one of Canada's largest generic-drug companies. And that's only if government regulators agree to fast-track their approval systems, he said, adding that the scope of the legislation is still up for debate and it's difficult to tell now how co-operative Canadian drug regulators will be"[43].

Civil Society was also a prominent voice but its major complaint was that Health Canada approval was not required by the WTO Decision and that it added an extra 6 months to the drug order. MSF argued that developing countries rely on drug approvals from the World Health Organization and that the Health Canada approval was superfluous and simply delayed things.

From the research-based industry, many statements came from Bayer Inc., which argued that safety was the main issue they requested to have moxifloxacillin taken off the list of eligible of medicines.

"We want to ensure that our products are used safely and appropriately in accordance with Health Canada's approved indications. When it comes to pharmaceutical therapies, surely patients in developing countries should not be subject to a lower standard of care than Canadians"[44].

The government's voice only issued a promise to fast-track its review of any products submitted for approval under the new law.

### Corruption, Diversion and Transparency

Some reports in the media generated concern over the diversion of medicines produced under CAMR back into developed country markets. Reports referred to the WTO requirements to prevent diversion, the potential for litigation if diversion occurred, and the implications of diversion.

"...generic knockoffs of AIDS drugs will 'leak' from the poor countries to other countries. Leaks can turn into floods and the cheap drugs could show up in richer countries where they would compete illegally with their brand-name versions. If that were to happen, the profits required to fund drug research and development programs could be threatened"[45].

Research-based industry voices were the most prominent, with one report concerned that the diversion of generic versions of their medicines would "undercut" the research-based industry's drugs sold in developed countries, "rendering patents worthless"[23]. Industry concerns appear to be focused on the detrimental effect these actions would have on the market value of medicines in developed world markets. The markets of Canada, the US and Europe are mentioned specifically.

The government was mainly silent on this issue but appeared to be equally concerned, with one government official paraphrased as saying:

"...in a worst-case scenario, drug manufacturers could even stop supplying Canada with certain drugs to fight AIDS and other health crises if the cheaper copies end up being sold in large numbers on the black market within Canada"[46].

The generic industry, on the other hand, was indicated as concerned that they would lose their license if diversion occurred. Civil society echoed their concerns.

One statement by an African NGO called for careful monitoring of imports into African countries with "less than transparent governments"[47].

"You have these cases where we get waivers [to import generics or to pay lower prices for patented medications] but people in power or working with drug companies take the opportunity to make money. Ministries of health are going to have to be careful"[47].

### Domestic Economy

The research-based industry's voice was the most dominant but only reflected initial statements by Harvey Bale saying that the introduction of this legislation would "hurt Canada as a destination for international research and development."

"It will be a 'negative black eye for Canada' that will 'very well affect the investment climate,' Mr. Bale said"[6].

In response, the government, whose voice was much less present on this issue, tried to comfort these concerns by reaffirming their commitment to protecting intellectual property because "innovation and knowledge is the future of Canada's economic success"[5].

Civil society was notably absent on this issue aside from a statement from Stephen Lewis which said that "a patent initiative would not undermine the domestic pharmaceutical industry"[4].

Other comments referred to the impact of HIV/AIDS on the domestic economies of developing countries, thereby threatening their political and social development.

### Industry Profits

Reports on the issue of profits appear to accept the idea that corporate entities respond to incentives and will not supply where there is no profit. These reports also seem to implicitly accept that the trade-off is between profit and health. One article does note the lack of market power in the countries that would benefit from CAMR.

Many voices were engaged on this issue. Most comments by civil society emphasized the need for financial incentives to engage the generic industry's participation and their concern about the litigation if firms charged prices higher than 25 per cent of the Canadian prices charged by the patent holder.

"'The scheme depends on generics using it -- essentially there has to be some economic incentive. Generics may have humanitarian reasons as well, but this can't just depend on goodwill. They are commercial enterprises,' says Elliott. 'For it to be economically worth their time and effort, they need economies of scale.'"[27].

Other comments framed the research-based industry as extremely profit-driven, expecting the industry to "fight long and hard to protect their profitable patents"[48].

Comments from the research-based industry, emphasized that Canadian generic shipments need to be non-profit or for humanitarian and non-commercial use only, linking it to the threat of litigation. Other statements spoke of the industry's efforts to provide their drugs at cost or below cost to parts of the developing world.

The generic industry counterbalanced the industry's concerns about profit saying that no business case could be made for exporting drugs at cost and that any CAMR initiatives would not be commercially significant for them. At most, they hoped "to gain profile, enhance its reputation for quality and build goodwill"[43].

"'It's an uncertain, difficult, lengthy process for our companies to basically sell below cost or donate products they don't even make [yet],' said Jeff Connell, spokesman for the Canadian Generic Pharmaceutical Association. 'It's unclear how it's all going to work out.'"[41].

During the drug order, Apotex spoke of its intention to charge MSF for only the price of the raw materials, while other reports claimed that MSF would pay 43 cents per pill for 150,000 pills, bringing in revenues of $64,500.

The Liberal government spoke only to defend the profit threshold of 25 per cent on the price, mainly on the basis of affordability and to respect trade obligations: "It keeps the fire to the feet of the generics"[49]. An industry official noted that some money could be made but that it did not accommodate lucrative transactions.

### Litigation

The generic drug industry was the most vocal in this category, arguing that CAMR's provisions expose them to many opportunities for litigation from the patent-holders, which would ultimately discourage them from participating in the initiative. They spoke of the litigious nature of the research-based pharmaceutical industry noting that they took Canadian generic companies to court approximately 320 times over the past decade.

"Mr. Connell argued brand-name drug companies are notoriously litigious, and cited as evidence their long record of suing the government of South Africa for infringing on drug patents. 'They sued Nelson Mandela,' he said. 'They won't think twice about taking our companies to court.'"[49].

Civil society supported these concerns, albeit at a much more muted level, saying that the provisions ultimately undermined the purpose of the legislation.

In contrast, research-based companies stated that they were unlikely to take legal action, as long as the generic companies maintained the non-profit nature of the initiative. The representative of Canada's Rx&D was quoted as saying that the generic companies were just making excuses for their unwillingness to participate in the initiative.

"'It seems the generics are spending a lot of energy on reasons why they can't do this,' spokesman Jacques Lefebvre said. 'As for being worried about lawsuits ... if you respect the regulations that are in place, you shouldn't worry about lawsuits.'"[41].

No statements from government were observed.

### Innovation

The media repeated the theme of a need for balance to be struck between the goal of innovation and the goal of access. Commentators both side with the argument of respecting patent rights, and also those who call for a new way to conduct business.

Those calling for respecting patent rights argued that the contrary would be the end of innovation. The brand industry reflected this claim, by citing their support for CAMR but emphasizing the importance of protecting IP, which they framed hand-in-hand with innovation. They emphasized the number of HIV/AIDS drugs and vaccines they had under development and at one point, suggested that the role of innovator might be more important than ensuring affordable generic drugs are available to the poor.

"Peter Bains, senior vice-president for Glaxo, noted that the company had already dramatically lowered the prices of its drugs (3TC, AZT and Combivir) and voluntarily granted Aspen a licence in 2001. He called yesterday's agreement 'an appropriate response to the HIV/AIDS pandemic in the region.' 'Perhaps more importantly, we are leading efforts to discover and develop new vaccines and medicines for AIDS,' he told reporters"[40].

The government clearly showed its value for innovation, through its economic importance as well as its health benefits, despite their comments at the early stages of the legislation that placed affordable drugs for developing countries as a priority.

"In the face of these realities, we cannot afford to conduct business as usual in the realm of foreign affairs. And frankly, we cannot afford to conduct business as usual in the realm of business, either. There are legitimate concerns that our access-to-medicines initiative might interfere with the capacity of pharmaceutical companies to fund the research and development that will produce new medicines of the future. We all benefit when intellectual property rights provide incentives for innovation. But the problem is that sick people in poor countries cannot wait until new medicines are affordable"[21].

### Production Costs

Few reports were made about whether Canada's generic industry would be able to produce drugs cheaply enough to win developing country contracts. In particular, concerns were raised late in the implementation stages about their ability to compete with other manufacturers in the developing world, given the low costs of production and raw materials.

The generic industry's voice appears to be the loudest, saying that the legislation makes it onerous and too costly to produce drugs under the legislation. Apotex stated that the price at which MSF had agreed to pay them for the drug wouldn't cover all of their research and legal expenses.

"'We've spent millions of dollars on the [research and development], we've spent lawyers' time at our cost, just because it's the right thing to do. It would be difficult to do again unless the legislation is made simpler,' Elie Betito said"[50].

There is some attention paid to the two-year contract limit and its effect on their viability for generics.

### Human Rights

Human rights was hardly mentioned in the media. The few statements that were made focused on the disparity between developing and developed countries. At the forefront of this discussion was Bill Graham in early November 2003, framing CAMR as a tool to achieve human rights and address the injustice.

"The current initiative sets a unique global standard on the frontiers of public health and human rights. For years there has been an injustice in the global response to diseases such as HIV/AIDS, tuberculosis and malaria: while prevention has been emphasized worldwide, only in developed countries have life-saving medicines been widely available. The result has been the sickness and deaths of millions, the devastation of communities, economies, and in some places the reversal of decades' worth of progress"[21].

## Discussion

Results provide insights into three aspects of the policy debate in the media: how stakeholders and the media framed the debate, which stakeholder's voice dominated the media debate and what the potential effects of media framing might be on the policy process and final policy product. Quantitative results show the range and emphasis of concepts that were raised by stakeholders. In large part, these policy goals are the language through which more fundamental policy disputes take place. Affordability, intellectual property, trade agreements and obligations, and development were the major issues raised but stakeholders interpreted these policy goals in very different ways.

Overall, the media conveyed different policy goals and trade-offs at the various stages of the policy's development and implementation. Initially, civil society called on the government to amend its patent law to save lives through production and export of cheap generic drugs at the expense of limiting intellectual property rights. The brand industry responded by framing CAMR as achieving nothing while trading off domestic R&D investment and eroding patent protection. Meanwhile, the Canadian foreign minister explicitly stated its intention to address drug affordability in poor countries despite the consequences such efforts have on intellectual property rights. The industry eventually supported the legislation; however, their emphasis on the need to comply with the WTO Decision signalled a shift in the nature of the debate. Subsequent government messages framed decisions that were unpopular with civil society and the generic industry as necessary in complying with WTO obligations. Civil society responded by framing their policy positions in similar terms.

By restricting the scope of issues up for discussion in debates, framing can hide the some of the assumptions that underpin the trade-offs underlying policy debates. The emphasis on intellectual property issues and trade agreements and obligations may seem obvious because CAMR is the implementation of the WTO Paragraph 6 Decision; however, these policy goals appeared to become ends in themselves in the media debates. Framing effectively obscured the debate from the bigger societal questions such as the role of intellectual property rights in drug innovation and affordability, the relationship between trade agreements and the domestic economy and whether policies like CAMR are even desirable in addressing the global drug inequity.

The topic of intellectual property law is a highly technical area, which makes it difficult to convey in real human terms the relevance and impact of WTO clauses under debate. In such contexts, simple rhetoric such as "breaking patents harm innovation" or "patents = death" holds appeal because it is comprehendible and believable. Actors use these sound-bites to push their specific policy points but these frames force stakeholders into defending their corners and hinder the way to finding common ground. In practice, the trade-offs in public policy are rarely certain and full of nuance, which does not find its way into a media focused on simple interpretations of a complex reality.

The frequency of the search terms also highlights what major issues were not mentioned. Drug production costs, developing country pressure, and procurement practices were hardly discussed but later identified by civil society as being fundamentally problematic within the regime[3]. Framing theory states that the emphasis some aspects of a problem naturally occurs at the expense of others[11]. Due to the focus on other issues such as drug quality, aid, industry profits and market competition, among others, little room was left to discuss these other implementation aspects of the regime.

Furthermore, the structure of the media policy debates left almost no space for the nature of Canada's human rights obligations to be examined. Early in the legislative process, the Liberal Government framed CAMR as a tool to promote human rights but this appears to have been empty rhetoric. The lack of discussion of human rights indicates a missed opportunity by all stakeholders to engage the public in a debate over how a country like Canada can make policy to realize these obligations in relation to drug access. It is possible that there was little institutional receptivity to human rights as a policy goal; however, introducing this aspect may have changed the nature and content of the policy debates.

The dominant voice across most categories which we examined was civil society. Their heavy use of the media, especially prior to the Canadian government's decision to introduce legislation, likely played an important agenda-setting role. Stephen Lewis amplified his call to the Canadian government to implement the WTO Decision through front page headlines, raising the public's hopes that Canada could play a leading role in the fight against AIDS. In contrast, civil society's influence on the policy contents may not have been as significant during the drafting of the legislation. The government addressed civil society's main complaint, the Right of Refusal, but CAMR retained several clauses that civil society, and the generic industry, claimed made the regime unworkable. Civil society's use of the media appears to have been skillful but it is possible that larger institutional, interest-based and political factors may have been at play which led to the outcome we see today. These factors are the subject of further research.

The lack of the brand industry's presence in the media is by no means an indicator of their interest in the legislation. Scope of conflict theory suggests that it is in the interests of the powerful to keep the conflict narrow in a policy debate[13]. It is possible that the brand industry did not have to rely on the news media to have its views incorporated into the legislative process.

Two interest groups that were notably less present or virtually absent from the media were generic companies and representatives from developing countries. The absence of the developing country voice is likely largely due to the limitation of our study to national newspapers, implying the heavy reliance on national sources of information. The generic industry's absence can be partly attributed to the fact that civil society held similar policy positions and already had a large presence in the media. More importantly, given the lack of potential profits from the legislation, their absence may have been a reflection of the generic industry's interest in seeing the legislation pass.

There are some limitations to our study. First, our qualitative analysis used a unique coding scheme, which limits the generalizability of our results. By providing the context for our analysis, readers will be able to apply the lessons learned from this case to other literature. Second, our analysis of actors' voices were not quantified, however in making our judgments, frequency was taken into account. Finally, the absence of analysis for episodic and thematic articles prevents us from making explicit inferences regarding the substantive nature of the debates, however our discussion was informed through an estimate of op-eds and letters analyzed in our data pool.

## Conclusions

This study illustrated how framing can restrict the policy debate, which has implications on the range of issues and policy alternatives considered. The lack of discussion of issues such as human rights obligations, pharmaceutical innovation and domestic economic issues suggests that some of the major underlying policy goals at stake in this case were not considered. Furthermore, the debate failed to highlight issues which are now posing to be significant barriers to the use of the legislation, namely drug production costs, procurement practices and pressure faced by developing countries in using similar patent law flexibilities. Using the media to engage the public in more in-depth exploration of the policy issues at stake may contribute to a more informed policy development process.

Civil society's use of the media appears to have been successful in adding pressure to the government to introduce the legislation, to remove the restrictive 'right of refusal' clause, and to initiate the legislative review during CAMR's implementation. In this sense, the media can be a venue in which those who do not have a strong voice in policy debates can leverage their position; however, the political and institutional context must be taken into account as it may outweigh media framing effects.

From this case, it appears that the media was a useful and effective channel for civil society to raise public attention to particular issues; however, its usefulness appears to be limited. Mass media is a relatively blunt instrument because framing effects can freeze the debate into particular perspectives, while limiting the nuanced debate needed for detailed, technical issues, which is where the problems with CAMR lie. CAMR was a hailed as a breakthrough concept by all stakeholders but problems arose during its implementation, and ultimately, the administrative and logistical requirements impeded the initiative. Parties with weaker influence may face limited benefit from the public pressure raised by media but the legislative process remains an important and effective forum to flesh out policy implementation details.

## Competing interests

LCE provided research support on Canada's Access to Medicines Regime while working as an unpaid intern for Médecins Sans Frontières' (MSF) in 2005 and as a paid consultant with the Canadian HIV/AIDS Legal Network and the North South Institute in April 2007.

## Authors' contributions

LCE participated in the conception of the study, developed the theoretical and coding framework, developed the qualitative analysis methods, participated in analyzing and interpreting the results, wrote the first draft of the manuscript and finalized the manuscript. KP participated in the conception of the study, contributed to the theoretical framework, designed the data collection strategy, participated in analyzing and interpreting the results and wrote the second draft of the manuscript. VK participated in the conception of the study, contributed to the coding framework, analyzed and interpreted the results, wrote the third draft of the manuscript and revised the manuscript critically for important intellectual content. AP participated in the conception of the study, contributed to the coding framework, managed the data, contributed to data analysis and interpretation of the results, wrote the fourth draft of the manuscript and revised the manuscript critically for important intellectual content. JCK participated in the conception and design of the study, contributed to interpreting the results and revised the manuscript critically for important intellectual content. All authors read and approved the final manuscript.

## Pre-publication history

The pre-publication history for this paper can be accessed here:

http://www.biomedcentral.com/1472-698X/10/1/prepub

## Supplementary Material

Additional File 1 **Summary of Policy Goals by Stakeholder Group**. A brief summary of the results grouped by policy goal and stakeholder is provided in table format. Click here for file
