# Supplementary material for: Framing access to medicines in developing countries: an analysis of media coverage of Canada's Access to Medicines Regime
Source: BMC Int Health Hum Rights. 2010 Jan 4;10:1. doi: 10.1186/1472-698X-10-1 (PMC2827390; doi:10.1186/1472-698X-10-1)
Supplement: Additional File 1 — Summary of Policy Goals by Stakeholder Group. A brief summary of the results grouped by policy goal and stakeholder is provided in table format. [file 1472-698X-10-1-S1.DOC]

Summary of Policy Goals by Stakeholder Group

| **Policy Goal** | **Stakeholder** | **Comments** |
| --- | --- | --- |
| **Affordability** | Canadian Government | Most frequently-heard voice on this issue (together with civil society). Government declared its commitment to making affordable drugs available to poor countries, with reports initially suggesting that Canada could become a major global supplier of affordable generic drugs. However, after the legislation passed, government voices heard less in regard to Canada's potential to contribute, saying that if the legislation simply encourages other countries to make more cheap drugs then it was fulfilling its goal. |
|  | Civil Society | Most frequently heard voice on this issue (together with government). Civil society viewed CAMR as part of the solution to the problem of affordable medicines in the developing world. Especially in regards to ARVs, civil society viewed CAMR as the key to generating greater price competition on global drug prices, given the prospect of existing generic drugs sources in India 'drying up'. |
|  | Research-Based Industry | Quoted infrequently on affordability, linking the legislation to the 'threat' of diversion of these medicines back into profitable, developed country markets. They also suggested that the issue had already been addressed, with India already supplying the developing world with AIDS medicines along with their own voluntary price reductions. |
|  | Generic Industry | Least vocal on this point. They referred mainly to the low cost of their drugs and their ability to compete globally. After the legislation was in effect, the generic industry mentioned the disinterest of Canadian generic companies in producing low cost drugs under the legislation, arguing as the brand industry did that the need was already being filled by suppliers from India. |
| **Intellectual Property** | Civil Society | Civil society voice was most dominant in this area. Emphasis was placed on the restrictions patent laws create to making drugs accessible to developing countries. Lewis appeared to echo Médecins Sans Frontières' rhetoric that patents were a "matter of life and death" and framed the Canadian government as holding Africa to a lower standard, given its move in 2001 to waive patent law to produce low-cost drugs for Canadian during the Anthrax scare. He framed CAMR as a quotient of hope for eliminating IP-related barriers to drug access. |
|  | Canadian Government | The Liberal Government was frequently observed on the issue of IP, framing it as innovation and knowledge, as providing incentives and funding for future R&D and for ensuring the development of new medicines for the future. One op-ed article, prior to the government's announcement, by Foreign Affairs Minister Bill Graham implied that in this particular case, drug affordability took precedence over patents. However, government clearly said that it still valued intellectual property protection, linking it to Canada's domestic economy. The government's 'balance' was framed as one between affordability and intellectual property rights, as well as international development and intellectual property rights. |
|  | Research-Based Industry | The research-based industry limited its remarks on IP but was clear on their position. Initially, the Director-General of the IFPMA, Harvey Bale, reacted with scathing criticism, linking the Canadian initiative to the erosion of intellectual property rights, which would negatively impact domestic R&D investment. Bale also accused the Canadian generic industry of ulterior motives to erode patent protection. After these initial statements, the research-based industry's tone changed immediately, citing support for the legislation and saying that Canada “has an opportunity to show international leadership” by changing patent laws to improve access to the drugs. The industry's position on IP was further evidenced through a letter to the editor suggesting that patents are not the barrier to drug access, citing that 95% of drugs listed on the WHO's Essential Medicines List are not protected by patents. |
|  | Generic Industry | The generic industry had virtually no comments aside from their reference to the [research-based] industry's litigious nature in relation to patent infringement. |
| **Development** | Civil Society | The dominant voice in this category was civil society, particularly Stephen Lewis and MSF. Their focus was on the terrible toll of HIV/AIDS on individuals and communities, framed in terms of emergency and human tragedy, as well as the fact that development in Africa is paralysed by the epidemic. They also emphasized the existing global drug inequity and the need for developed countries to support Africa by making drugs available. |
|  | Canadian Government | The government framed CAMR largely as a tool to help address the HIV/AIDS pandemic and therefore address development, with the Prime Minister and Minister of International Trade particularly supportive of the initiative. The Liberal government's initiatives and financial commitments to HIV/AIDS programming and social development initiatives were mentioned |
|  | Research-Based Industry | The research-based industry framed development as part of the problem of drug access and therefore it should be first addressed through improved health care infrastructure, training and delivery. Upon announcement of the legislation, they argued that access to cheaper generic drugs should be at the “bottom of the totem pole” of solutions and that cash should be funnelled into the infrastructure of developing countries and to the Global Fund. |
|  | IGO | WTO Director General is reported to have supported the right of poor countries to make full use of TRIPS flexibilities to counter diseases, while a research-based industry op-ed piece quoted the WHO Director General as saying that better health infrastructure and clean water are essential to effectiveness of medicines. |
|  | Generic Industry | The generic industry was notably silent on this issue. |
|  | Developing Country Representatives | Hardly mentioned were calls to develop manufacturing capacity in the developing world and inadequate institutional capacity to implement the WTO provisions, the latter noted by a Tanzanian government official during the 2006 AIDS Conference. |
| **Aid** | IGO | Stephen Lewis, in his capacity as UN Special Envoy, spoke of the massive inflow of aid and antiretroviral drugs required in sub-Saharan Africa. He framed this issue in terms of the West’s moral deficit, criticized Canada specifically for lagging in its aid commitments and framed CAMR as a way to restore some of Canada's “moral authority”. |
|  | Generic Industry | The generic industry is reported as supporting CAMR, framing it as an aid initiative, but having a problem with its provisions for litigation. |
|  | Canadian Government | The Liberal Government is reported as being enthusiastically supportive of initiative to make cheap drugs more available. Paul Martin reported to go further than that and urged other G7 countries to donate to the Global Fund, assist developing countries to win approval from WTO to import cheap drugs and to exploit CIDA's capacity. |
|  | Research-Based Industry | The research-based industry indicated full support of the initiative, citing the need to improve access to medicines and health care systems. In an opinion piece, the industry recognized the human toll caused by HIV/AIDS. They established that its commitment to this cause predates CAMR, by enumerating its monetary donations, partnerships with NGOs, and capacity building in Africa. |
| **Market competition** | Civil Society | Civil society was the dominant voice on this issue, arguing that CAMR's purpose is to increase generic competition in the market for antiretroviral drugs. They criticized CAMR's "right of refusal" clause as giving the research-based industry the opportunity to block generic competition needed to bring prices down and criticized the research-based industry for "monopolistic" behavior. |
|  | Developing Country Representatives | Some activists in the African community viewed CAMR as capable of producing a significant downward effect on global drug prices. |
|  | Research-Based Industry | The research-based industry argued that the "right of refusal" clause was competitive and efficient. They also argued that the global market had no room for Canadian generic companies since they could not compete against the Indian and Brazilian generic drug companies. |
|  | Generic Industry | Generic companies seemed to acknowledge the possibility that they may not be able to compete globally on price but that they would lead globally on quality for antiretroviral drugs. |
| **Trade Agreements and Obligations** | Civil Society | Civil society was the most noticeable voice in this category. Early on, they called on the government to implement the WTO Paragraph 6 Decision, arguing that if "robustly implemented", it would be worthwhile. During the legislative process, civil society framed CAMR as going above and beyond what was required by the WTO Decision. After CAMR was passed, NGOs commonly linked the flaws in the system to the original WTO framework. They consistently emphasized the threat of trade and intellectual property laws on access to medicines. |
|  | Government | Government's voice was less pronounced on this issue, emphasizing their leadership on the implementation of the WTO Agreement and hoping their legislation would become a model. As the legislative process unfolded, they emphasized the complexities and ambiguities of the WTO Agreement, showing trepidation. |
|  | Research-Based Industry | The research-based industry argued that the WTO Decision should be implemented taking into account future innovation and the development needs of developing countries. |
|  | Generic Industry | The generic industry was mostly silent until CAMR passed. During implementation stage, the generic industry blamed the complexity of the WTO Agreement for lack of results and on CAMR's additional and unnecessary requirements. |
| **Developing Country Pressure** | Civil Society | Civil society was almost the only voice on this issue, arguing that developing countries face political pressure against using TRIPS flexibilities to improve access to medicines. The WTO Paragraph 6 Decision offers opportunities for this political pressure. |
|  | Developing Country Representatives | The Tanzanian High Commissioner was quoted saying that countries might face political pressure not to use CAMR or the WTO Paragraph 6 Decision. |
| **Quality, Safety and Regulatory Approval** | Generic Industry | The generic industry's voice was most prominent, framing their role as setting a standard for the quality of generic AIDS drugs. Apotex suggested the "lengthy" drug approval process, even if fast-tracked, as an additional disincentive to generic participation. |
|  | Civil Society | Civil society argued that Health Canada approval was superfluous and simply delayed the process. |
|  | Research-Based Industry | Bayer, Inc. argued that their requests to have moxifloxacillin removed from the list of eligible medicines were based upon safety concerns. |
|  | Government | The government hardly mentioned anything aside from a promise to fast-track its review of products under CAMR. |
| **Corruption, Diversion and Transparency** | Research-based industry | The research-based industry was the most dominant voice, with one report suggesting that the diversion of generic versions of their medicines would "undercut" their drugs "rendering patents worthless". |
|  | Government | The government issued one statement warning that diversion could lead to the withdrawal of some drugs off the Canadian market by the research-based industry. |
|  | Generic Industry | The generic industry was concerned that they would lose their compulsory license if diversion occurred. |
|  | Civil Society | Civil society was concerned that they would lose their compulsory license if diversion occurred. |
|  | Developing Country Representatives | A representative from an African NGO warned about the potential for corruption by developing country governments. |
| **Domestic Economy** | Research-Based Industry | The research-based industry's voice was most dominant, reflecting early statements by the IFPMA that CAMR would "hurt Canada as a destination for international research and development". |
|  | Government | The government's voice was much less present on this issue. They linked Canada's economic success to protecting intellectual property to encourage innovation and knowledge. |
|  | Civil Society | Civil society was noticeably absent on this issue aside from Stephen Lewis' suggesting that CAMR would not undermine the domestic pharmaceutical industry. |
| **Industry Profits** | Civil Society | Civil society was among many voices on this issue. They emphasized the need for financial incentives to engage the generic industry's participation and that CAMR could not rely on goodwill. They were concerned about potential litigation if the contract was deemed commercial in nature. They framed the research-based industry as extremely profit-driven. |
|  | Research-Based Industry | The research-based industry was equally vocal on this topic, arguing that Canadian generic shipments had to be non-profit, linking it to the threat of litigation. They spoke of their aid programs providing non-profit drugs to the developing world. |
|  | Generic Industry | The generic industry was equally vocal on this topic, stating that no business case could be made for exporting drugs at cost and that CAMR would not be commercially significant for them. |
|  | Government | The Liberal government spoke only to defend the profit threshold of 25 percent on the price, mainly on the grounds of affordability and to respect trade obligations. |
| **Patent Litigation** | Generic Industry | The generic industry was the most vocal in this category, arguing that CAMR's provisions expose them to many opportunities for litigation from patent-holders, which would ultimately discourage them from participating in the initiative. |
|  | Civil Society | Civil society support the generic industry's concerns, albeit at a more muted level, saying that the provisions ultimately undermined the purpose of the legislation. |
|  | Research-Based Industry | Research-based companies stated that they were unlikely to take legal action, as long as the generic companies maintained the non-profit nature of the initiative. |
| **Innovation** | Research-Based Industry | The research-based industry cited their support for CAMR but emphasized the importance of protecting intellectual property, which they framed hand in hand with innovation. They emphasized the importance of innovation alongside and potentially above, drug affordability. |
|  | Government | In the early stages of the legislative process, the government appeared to place the goal of affordable drugs for developing countries as a priority over innovation, while still expressing value for innovation through its economic importance and to curing disease. |
| **Production Costs** | Generic Industry | The generic industry's voice was most prominent, saying that the legislation made it onerous and too costly to produce drugs under the legislation. |
| **Human Rights** | Government | In the early legislative stages, the government framed CAMR as a tool to achieve human rights and address the injustice of the lack of access to medicines. |
